# CircNRIP1 Encapsulated by Bone Marrow Mesenchymal Stem Cell–Derived Extracellular Vesicles Aggravates Osteosarcoma by Modulating the miR-532-3p/AKT3/PI3K/AKT Axis

**DOI:** 10.3389/fonc.2021.658139

**Published:** 2021-09-29

**Authors:** Zuowei Shi, Kaifu Wang, Yufei Xing, Xuefeng Yang

**Affiliations:** Department of Orthopaedics, First Hospital of Harbin Medical University, Harbin, China

**Keywords:** osteosarcoma, bone marrow mesenchymal stem cells, extracellular vesicles, circular RNA NRIP1, microRNA-532-3p, AKT3, PI3K/AKT signaling pathway

## Abstract

Emerging evidence indicates that extracellular vesicle (EV)-encapsulated circRNAs have the potential diagnostic and prognostic values for malignancies. However, the role of circNRIP1 in osteosarcoma remains unclear. We herein investigated the therapeutic potential of circNRIP1 delivered by bone marrow mesenchymal stem cell–derived EVs (BMSC-EVs) in osteosarcoma. The expression of circNRIP1 was examined in the clinical tissue samples of osteosarcoma patients, after which the downstream genes of circNRIP1 were bioinformatically predicted. Gain- and loss-of function assays were then performed in osteosarcoma cells with manipulation of circNRIP1 and miR-532-3p expression. EVs isolated from BMSCs were characterized and co-cultured with osteosarcoma cells to examine their effects on cell phenotypes, as reflected by CCK-8 and Transwell assays. Further, a mouse model of tumor xenografts was established for *in vivo* substantiation. circNRIP1 was upregulated in osteosarcoma tissues and cells. Overexpression of circNRIP1 promoted the proliferative, migratory, and invasive potential of osteosarcoma cells. Co-culture data showed that BMSC-EVs could transfer circNRIP1 into osteosarcoma cells where it competitively bound to miR-532-3p and weakened miR-532-3p’s binding ability to AKT3. By this mechanism, the PI3K/AKT signaling pathway was activated and the malignant characteristics of osteosarcoma cells were stimulated. *In vivo* experimental results unveiled that circNRIP1-overexpressing BMSC-EVs in nude mice resulted in enhanced tumor growth. In conclusion, the BMSC-EV-enclosed circNRIP1 revealed a new molecular mechanism in the pathogenesis of osteosarcoma, which might provide a novel therapeutic target for osteosarcoma.

## Introduction

Osteosarcoma represents the most common primary malignant bone tumor in children and adolescents, showing a very heterogeneous genetic profile (1). Currently, osteosarcoma treatment is based on systemic chemotherapy, immunotherapy, and surgery (2), but the overall clinical outcomes of osteosarcoma patients remain unsatisfactory (3). Herein, a better understanding of the processes that drive osteosarcoma progression is critical for the development of novel therapeutic targets.

Bone marrow–derived mesenchymal stem cells (BMSCs) are non-hematopoietic stem cells with multi-differentiation potentials, and BMSCs are the most commonly used stem cells in aspects of cell therapy and tissue repair (4, 5). Notably, extracellular vesicles (EVs) derived from BMSCs have been implicated in the progression of several cancers such as lung cancer (6), cervical cancer (7), and myeloma (8). As a promising cell-free therapy, EVs function through shuttling bioactive cargoes such as proteins, RNAs, mRNAs, microRNAs (miRNAs), lipids, and organelles to recipient cells and have higher safety profile (9). Further, lymphocyte cytosolic protein 1 shuttled by BMSC-derived exosomes has been reported to promote cell proliferation and metastasis in osteosarcoma (10), while BMSC-derived exosomal miR-206 has been highlighted to impede osteosarcoma progression (11), indicating the promising role of EVs derived from BMSCs in the management of osteosarcoma.

Circular RNAs (circRNAs), a type of non-coding RNAs, have been found aberrantly expressed in multiple diseases and play a pivotal role in cancer initiation, progression, and immune response (12). Of note, accumulating evidence has unraveled that circRNAs participated in multiple aspects of osteosarcoma development, such as osteosarcoma cell metastasis (13), drug resistance (14), and *in vivo* tumorigenesis (12), on the basis of their biological functions as miRNA sponge, gene regulators at posttranscriptional levels, and also interactors with proteins (15, 16). Further, circNRIP1 (also known as hsa_circ_0004771) has been documented to be a novel diagnostic biomarker and an oncogenic circRNA for malignant development in several cancers, including gastric cancer and colorectal cancer due to its differential expression in these cancers (17, 18). Following previous documentation, we thereby explore in the present study whether circNRIP1 also has a role to confer in osteosarcoma. The StarBase database predicted the presence of complementary binding sites between circNRIP1 and miR-532-3p. miRNAs, a class of non-coding RNA molecules, have critical roles to confer in the progression of a wide spectrum of cancers (19). Specifically, miR-532-5p has been previously documented as a prognostic marker of osteosarcoma owing to its ability to inhibit the malignant phenotype of osteosarcoma by targeting chemokine ligand-2 (CXCL2) (20).

Furthermore, miR-532-3p binding sites in the 3’-untranslated region (3’-UTR) of AKT3 mRNA was predicted by the StarBase database. AKT3 shows an evident upregulation in osteosarcoma tissues and cells, and this upregulation potentiates cell proliferation as well as inhibiting cell cycle arrest at the G0/G1 phase (21). The phosphoinositide 3-kinase (PI3K) lipid signaling pathway is well-recognized to modulate almost all cellular behaviors involved in growth and normal physiology (22). Aberrant PI3K signaling has been regarded as a hallmark of a variety of diseases, correlated with the regulation of downstream effectors; a most extensively studied effector is the protein kinase AKT, which in mammals is comprised of three isoforms, namely, AKT1, AKT2, and AKT3 (23). The PI3K/AKT signaling pathway represents a vital intracellular signaling pathway associated with various cellular functions essential for survival and growth in normal physiological conditions and in pathological disorders (24). Activation of the PI3K/AKT signaling pathway contributes to promotion of the proliferation and metastasis of osteosarcoma cells (25).

We therefore proposed a hypothesis that circNRIP1 delivered by bone marrow mesenchymal stem cell–derived EVs (BMSC-EVs) may augment osteosarcoma development involving the interaction with miR-532-3p, AKT3, and PI3K/AKT. To address this hypothesis, we isolated EVs from BMSCs and conducted co-culture experiments so as to provide potential therapeutic strategies against osteosarcoma.

## Materials and Methods

### Ethics Statement

The current study was performed with the approval of the Ethics Committee of First Hospital of Harbin Medical University and in strict accordance with the *Declaration of Helsinki*. All participants or caregivers signed informed consent documentation. Animal experiments were approved by the Animal Ethics Committee of First Hospital of Harbin Medical University and performed according to the Guide for the Care and Use of Laboratory Animals published by the US National Institutes of Health.

### Microarray-Based Gene Expression Profiling

Osteosarcoma-related gene expression dataset GSE41445 was retrieved from the GEO database, which contained three normal samples and three tumor samples. Differential analysis was conducted utilizing R language “limma” package with |logFC| > 2 and false discovery rate (FDR) < 0.05 set as the threshold. The obtained genes were then subjected to KEGG enrichment analysis utilizing R “clusterprofiler” package. Relevant information of circNRIP1 was retrieved utilizing the circBase database (http://www.circbase.org/). The StarBase database (starbase.sysu.edu.cn/) was applied to predict the upstream miRNA of AKT3. The downstream miRNA of circNRIP1 was predicted by the circInteractome database (https://circinteractome.nia.nih.gov/).

### Sample Collection

A total of 30 patients (18 males and 12 females, aged 12–26 years with an average age of 18.5) diagnosed with primary osteosarcoma at First Hospital of Harbin Medical University from 2015 to 2018 were enrolled in the current study. All patients underwent complete resection without preoperative chemoradiotherapy. According to the Enneking staging system, 6 cases were at stage 1, 17 cases at stage 2, and 7 cases at stage 3. All patients were diagnosed through the combination of bone marrow cell morphology, flow cytometry, and histopathological evaluation of bone marrow biopsy specimens. None of the participants underwent surgery or chemotherapy prior to the enrollment, and those with a history of other chronic diseases were excluded. Normal tissues adjacent to the tumor (more than 5 cm away from the edge of tumors) were harvested as the control, which were identified to be normal in morphology by two independent pathologists. All resected or biopsy specimens were immediately frozen in liquid nitrogen and stored at −80°C for subsequent experiments.

### Cell Culture and Treatment

Human osteoblast cell line hFOB 1.19 (GNHu14) and osteosarcoma cell lines MG63 (TCHu124) and U2OS (SCSP5030) were purchased from the cell bank of Chinese Academy of Sciences (Shanghai, China). These cells were cultured in Dulbecco’s modified Eagle’s medium (DMEM) containing 10% FBS, 100 U/ml penicillin, and 100 μg/ml streptomycin in a 5% CO_2_ incubator at 37°C.

Lentiviral particles carrying circNRIP1 overexpression plasmids (oe-circNRIP1) or small interfering RNA (siRNA) targeting circNRIP1 (si-circNRIP1) or corresponding negative control (oe-NC, si-NC) were constructed in HEK-293T cells utilizing lentiviral package kit (Invitrogen, Carlsbad, CA, USA). After 48 h, the virus-containing supernatant was collected, and the virus was concentrated by Shanghai Genechem Co., Ltd. (Shanghai, China). Upon reaching 50% density, U2OS cells, MG63 cells, and BMSCs were transduced with lentivirus and, 48 h later, incubated with 10 μg/ml puromycin (Sigma-Aldrich, St. Louis, MO, USA) for at least 1 week to screen out stably transfected cell lines. Considering the off-target effect, multiple siRNAs targeting different sequences were designed, and the one with the optimal silencing efficiency was selected for subsequent experiments.

miR-532-3p mimic, miR-532-3p inhibitor, and the corresponding NCs were purchased from Genechem. Osteosarcoma cells were seeded in a six-well plate at a density of 4 × 10^5^ cells/ml. When reaching approximately 80% confluence, the cells were transfected according to the instructions of Lipofectamin 2000 reagents (11668-019, Invitrogen). The cells were further incubated for 48 h for subsequent experiments. For additional LY294002 (a specific inhibitor of PI3K) treatment, MG63 cells were added with LY294002 (50 μM; HY-10108, MedChemExpress, Monmouth Junction, NJ, USA) after 24-h transfection and then incubated for 24 h.

### Characterization of BMSCs by Flow Cytometry

The BMSCs at passage 3 with approximately 80% confluence were selected for identification. Initially, the culture medium was removed, and the precipitate after centrifugation was digested. Following two washes with PBS, the cells were counted with the density adjusted to 1 × 10^6^ cells/ml, and transferred into a 15 ml centrifuge tube with 100 μl PBS buffer containing 2% FBS. Cells were incubated with specific fluorescent flow cytometry antibodies against CD90, CD105, and CD44 (1:100, all were phycoerythrin [PE]-labeled antibodies from Biolegend, San Diego, CA, USA) or CD34, CD45, and CD14 (1:100, all were fluorescein isothiocyanate [FITC]-labeled antibodies from BD Biosciences, San Jose, CA, USA) at 4°C for 30 min in the dark. After re-suspension, cells were centrifuged and then added with 300 μl PBS buffer; while the background marker in the control group was determined by homotype monoclonal antibody. The fluorescent cells were analyzed by flow cytometry (BD FACSVerse). The positive rate of surface antigen was calculated utilizing FlowJo software (FlowJo, LLC, Ashland, OR, USA), with the unit of %.

### Isolation and Identification of EVs

FBS was ultracentrifuged at 100,000 g for 18 h to remove EVs from serum, and BMSCs were incubated in DMEM medium supplemented with 10% EV-free FBS. When BMSCs reached approximately 80% confluence, the supernatant of culture medium was removed, and 10% EV-free FBS culture medium was replaced, followed by culture in a 5% CO_2_ incubator at 37°C for 48 h. The obtained supernatant was centrifuged at 500 g and 4°C for 15 min to remove the cell fragments, and additional centrifugation was conducted at 10,000 g and 4°C for 20 min to withdraw large vesicles. Following filtration through a 0.22-micron filter, the cells were centrifuged at 110,000 g for 70 min, and then resuspended in PBS at 4°C. Next, the cells were ultracentrifuged at the same conditions and resuspended with 100 μl sterile PBS. All ultracentrifugations were conducted with Beckman ultracentrifuge (Beckman-Coulter Optima L-90k Ultracentrifuge, Type 45Ti rotor) at 4°C. The low-speed centrifugation was performed by Beckman Allegra X-15R table centrifuge. Nanoparticle tracking analysis (NTA) with a NanoSight nanoparticle tracking analyzer (Malvern Instruments, Malvern, UK) was used to measure the size distribution of EVs. The morphology of EVs was then observed under a transmission electron microscopy (TEM; H7650, Hitachi, Japan). The expression of EV-specific surface markers [TSG101 (ab30871, Abcam), CD81 (ab92726, Abcam), ALIX (ab76608, Abcam), APOB (ab184990, Abcam), and GRP94 (ab3674, Abcam)] was detected utilizing western blot analysis to identify the characteristics of EVs.

### BMSC-EV-circNRIP1 Content Measurement

RNase A treatment was conducted to determine whether the circRNA was surface bound or packaged in EVs. In short, EVs were resuspended in PBS and incubated with 20 μg/μl RNase A (Purelink RNase A, Life technologies, Gaithersburg, MD, USA) for 20 min at 37°C. Next, the integrity of vesicle membrane was destroyed by detergent treatment to determine the specificity of circRNA cargo. RNase A was used for the above treatment, and RIPA buffer was added to the EVs and allowed to stand for 20 min to terminate the RNase A activity reaction. The content of circNRIP1 was then determined utilizing RT-qPCR.

### Confocal Microscopic Observation and Quantification of circNRIP1-Loaded BMSC-EVs Internalized by Osteosarcoma Cells

The EVs extracted from BMSCs were labeled with the red dye Dil (Invitrogen), washed in serum-free medium, and resuspended. hFOB 1.19, MG63, and U2OS cells were seeded on a single-layer glass chassis (Cellvis, Mountain View, CA, USA) and then co-cultured with Dil-labeled EVs. Following PBS washing, the cells were fixed in 4% paraformaldehyde for 10 min, stained with FITC-circNRIP1 probe, and then observed with a confocal microscope (Leica TCS SP8).

Further, the osteosarcoma cell lines MG63 (TCHu124) and U2OS (SCSP-5030) were classified into four groups and subjected to different treatments: the control group (untreated cells), the BMSCs-EVs group [cells co-cultured with EVs isolated from BMSCs conditioned medium (CM)], the BMSCs-CM group (cells co-cultured with the same volume of BMSCs CM), and the BMSCs-EVs + GW4869 group [cells co-cultured with EVs isolated from BMSCs culture medium in the presence of EV secretion inhibitor GW4869 (HY-19363, MedChemExpress)]. Subsequently, RT-qPCR was performed to detect the expression of circNRIP1 in each group, and the primers were shown in **Supplementary Table 1**.

### RNA Isolation and Quantitation

The total RNA was extracted from cells and tissues with TRIzol reagents (15596-018, Solarbio, Beijing, China). For the detection of mRNA, the extracted RNA was reverse transcribed into complementary DNA (cDNA) utilizing the the PrimeScript™ RT-PCR kit (TaKaRa, Mountain View, CA, USA). For the detection of miRNA, the extracted RNA was reverse transcribed into cDNA utilizing the PrimeScript™ miRNA RT-PCR kit (B532451, Sangon Biotech, China). RT-qPCR was conducted utilizing SYBR Premix Ex TaqTM (TaKaRa) on LightCycler 480 system (Roche Diagnostics, Pleasanton, CA, USa). The primers were designed by Shanghai General Biotechnology Co. Ltd. (Shanghai, China) (**Supplementary Table 1**). Human mRNA and miRNA were normalized to GAPDH and U6, respectively, while mouse mRNA and miRNA were normalized to β-actin. The fold changes were calculated utilizing the 2^−ΔΔCt^ method.

### Western Blot Analysis

The total protein was extracted from cells with enhanced RIPA buffer (Boster Biological Technology Co., Ltd., Wuhan, Hubei, China) with protease inhibitor, followed by concentration determination by a bicinchoninic acid (BCA) kit (Boster). The protein was then separated utilizing 10% sodium dodecyl sulfate polyacrylamide gel electrophoresis and transferred onto polyvinylidene fluoride membranes. The membranes were then blocked utilizing 5% bovine serum albumin (BSA) at room temperature for 2 h and underwent overnight incubation at 4°C with diluted primary antibodies including rabbit anti-human and mouse AKT3 (ab179463, 1:1,000: Abcam, Cambridge, UK), p-AKT (ab38449, 1:1,000; Abcam), p-mTOR (ab109268, 1:1,000; Abcam), and mTOR antibody (ab134903, 1:10,000; Abcam). The next day, the membranes were incubated with secondary antibody goat anti-rabbit IgG (ab6721, 1: 5,000, Abcam). Band intensities were quantified utilizing Image J software. The ratio of the gray value of the target band to GAPDH (ab9485, 1:2,500, Abcam) was representative of the relative protein expression.

### Cell Counting Kit-8 Assay

Cells were seeded into a 96-well plate at a density of 5 × 10^3^ cells/well. Next, 10 µl of CCK-8 solution (Dojindo Laboratories, Kumamoto, Japan) was added to each well and incubated at 37°C for 1–3 days. The optical density (OD) value of each well was measured utilizing a microplate reader (Thermo Fisher Scientific Inc., Waltham, MA, USA) at 450 nm.

### Transwell Assay

Osteosarcoma cells were seeded in six-well plates and incubated for 24 h, with the medium renewed. For the EV treatment, BMSCs-EVs (50 μl of exosomes, 0.8 μg/μl) or the same volume of PBS was added to the cell culture medium of each group, followed by another 24-h incubation, and the Transwell assay was then performed.

Migration assay was conducted as follows: after 24 h, the cells were digested to a final concentration of 2 × 10^5^ cells/ml. Then 0.2 ml of suspension was added to the upper Transwell chamber, and 700 μl pre-cooled DMEM containing 10% FBS was added into the lower chamber. After 24 h, the cells were incubated in a 5% CO_2_ incubator at 37°C, and 24 h later, they were transferred to the Transwell chamber, fixed with methanol, and stained with 0.1% crystal violet.

Invasion assay was conducted as follows: before the experiment, 50 μl of Matrigel (Sigma-Aldrich) was put on the chamber to coat the filter membrane, and osteosarcoma cells were seeded in the upper chamber. The remaining procedures were the same as above. The number of stained cells was counted under an inverted microscope (XDS-800D, Shanghai Caikon Optical Instrument Co. Ltd., Shanghai, China). Five visual fields were randomly selected for counting, and the number of cells was expressed as the mean value.

### Dual-Luciferase Reporter Assay

The binding relationship between miR-532-3p and AKT3 was predicted utilizing the RNA22 database, which was further confirmed by conducting dual-luciferase reporter assay. The AKT3 3’-UTR sequence containing the predicted miR-532-3p binding site was inserted into the pGL3-basic vector (Promega Corporation, Madison, WI, USA) of XbaI restriction site downstream of luciferase gene to generate firefly/renilla luciferase reporter vector pGL3-basic-AKT3-3’-UTR-wild type (WT). The mutant was pGL3-basic-AKT3-3’-UTR-mutant type (MUT). Sequences of AKT3-3’-UTR-WT and AKT3-3’-UTR-MUT were listed in **Supplementary Table 2**. The reporter plasmids were then co-transfected with miR-532-3p mimic and NC-mimic plasmids into HEK-293T cells. After 24 h, the cells were harvested and lysed, followed by lysate collection. Dual-Luciferase^®^ Reporter Assay System (E1910, Promega) was used to detect the luciferase activity, which was normalized to the renilla luciferase activity.

### Fluorescence *In Situ* Hybridization

Osteosarcoma cells were seeded in six-well plates. Upon reaching 60–70% confluence, the cells were fixed in 4% formaldehyde for 10 min and permeated with 0.5% Triton X-100 in PBS at 4°C for 15 min. The cells were then incubated with FITC-labeled miR-532-3p probe at 37°C overnight and then washed six times with 2× saline sodium citrate (SSC) (3 min per wash). Next, Cy3-labeled AKT3 probe and pre-hybridization buffer (1:100) were incubated with cells at 88°C for 3–5 min, followed by another incubation at 4°C for 3 min. Thereafter, the cells were incubated with Cy3-labeled AKT3 probe at 37°C overnight and washed six times with 2× SSC (3 min per wash), followed by cell staining utilizing 4’, 6-diamidino-2-phenylindole (DAPI). Five fields of view were selected and observed by fluorescence microscopy (Olympus Optical Co., Ltd, Tokyo, Japan).

### RNA Pull-Down Assay

The collected cells were lysed and treated with ultrasound. circNRIP1 probe was incubated with C-1 magnetic beads (Life Technologies) at 25°C for 2 h to generate probe-coupled magnetic beads. Cell lysates were incubated at 4°C overnight with circNRIP1 probe or oligonucleotide probe. The following day, the RNA was washed, eluted, and purified, after which it was analyzed by RT-qPCR to detect the enrichment of miR-532-3p.

### Xenograft Tumors in Nude Mice

A total of 24 4-to-6-week-old male BALB/c nude mice (nu/nu; Beijing Animal Center, Chinese Academy of Medical Sciences, Beijing, China) were housed at specific pathogen-free environment at 26–28°C and 50–65% humidity. The mice were classified into threee groups (n = 8) and accordingly injected with 50 μl (1 μg/μl) of BMSC-EVs-enclosed with different cargoes (si-circNRIP1, oe-circNRIP1, and Vector; sequences shown in **Supplementary Table 3**) though tail vein the next day after subcutaneous inoculation of MG63 cells (5 × 10^6^ cells) in the left axillary region. From day 7 after the inoculation, the weight of mice was measured every 7 days, and the growth of tumor was observed and photographed. Five weeks after inoculation, the mice were euthanized by cervical dislocation. The tumor tissue was extracted, and the weight was measured with a balance. The expression of circNRIP1, AKT3, and miR-532-3p in tumor tissues was determined.

### Statistical Analysis

All data were analyzed utilizing SPSS 21.0 statistical software (IBM Corp. Armonk, NY, USA). The measurement data were described as mean ± standard deviation. Data between osteosarcoma tissues and adjacent normal tissues were compared utilizing paired *t*-test and those between the other two groups by unpaired *t*-test. Differences among multiple groups were statistically analyzed employing one-way analysis of variance (ANOVA) and Tukey’s multiple comparisons test. Pearson’s correlation coefficient was used for correlation analysis. Statistical analysis in relation to time-based measurements within each group was realized utilizing repeated measures ANOVA, followed by Bonferroni *post hoc* test for multiple comparisons. A value of *p* < 0.05 was statistically significant.

## Results

### miRNA and mRNA Expression Profiles in Osteosarcoma

**Figure 1:**
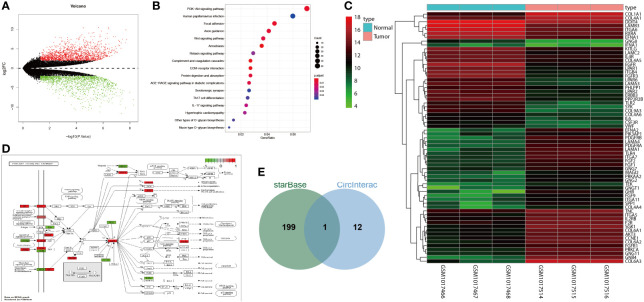
circNRIP1 might be involved in the osteosarcoma development *via* alteration of miR-532-3p and AKT3. **(A)** A volcano of differentially expressed genes analyzed by the osteosarcoma-related GSE41445 dataset. The abscissa represents -log10 *p* value, and the ordinate represents log2FC; each point in the graph represents a gene in which the red point represents significantly highly expressed genes in osteosarcoma, and the green point represents significantly poorly expressed genes in osteosarcoma. **(B)** KEGG enrichment analysis of differentially expressed genes. The abscissa represents GeneRatio, and the ordinate represents KEGG entry; the circle size in the graph represents the number of genes enriched in the entry, and the color represents *p* value. **(C)** A volcano of differentially expressed genes in the PI3K/AKT signaling pathway. The abscissa represents sample number, and the ordinate represents gene name; the left dendrogram represents gene expression cluster, each small square represents the expression of a gene in a sample, and the upper right histogram is color scale. **(D)** Labeling of differentially expressed genes in the PI3K/AKT signaling pathway. Green box represents significantly poorly expressed genes in osteosarcoma, and red box represents significantly highly expressed genes in osteosarcoma. **(E)** Intersection of AKT3 upstream regulatory miRNAs and circNRIP1 downstream regulatory miRNAs. The central part represents the intersection of two groups of data.

Differential analysis of the osteosarcoma-related GSE41445 dataset revealed 2,743 differentially expressed genes (**Figure 1A**). Further KEGG enrichment analysis showed that these differentially expressed genes were mainly enriched in the PI3K/AKT signaling pathway (**Figure 1B**). In the PI3K/AKT signaling pathway, 65 differentially expressed genes were enriched (**Figure 1C**). Then, the location of these genes in the signaling pathway was labeled with the results showing that AKT3 was at the core, and it was highly expressed in osteosarcoma (**Figure 1D**).

StarBase database predicted 200 candidate AKT3 upstream regulatory miRNAs. circNRIP1 has been shown to be expressed in BMSCs and could augment the development of various tumors (26, 27). The circInteractome database predicted the downstream miRNAs of circNRIP1, which were then subjected to intersection analysis with the predicted AKT3 upstream miRNAs. The results yielded only one candidate miRNA miR-532-3p (**Figure 1E**). These results suggested that circNRIP1 may affect the occurrence and development of osteosarcoma through the miR-532-3p/AKT3 axis.

### circNRIP1 in BMSCs Can Be Delivered by EVs to Osteosarcoma Cells

Flow cytometry was used to detect the expression of classic surface markers of BMSCs. As depicted in **Supplementary Figure 1A**, CD63, CD90, CD44, and CD105 showed positive expression rate higher than 95%, while CD34, CD45, and CD14 showed positive expression rate lower than 2%. This indicated that BMSCs had high purity and could be used for subsequent experiments. The morphology of BMSCs was analyzed under a light microscope where BMSCs appeared long fusiform or spindle, and grew in a manner of colony. When cells were dense, they were arranged like a vortex (**Supplementary Figure 1B**). At the same time, EVs were isolated from BMSCs and then identified. TEM observation showed that the EVs had double membranes, and moreover, the size was in the range from 30 to 120 nm, analyzed by NTA (**Supplementary Figures 1C, D**). Western blot analysis further demonstrated that the ALIX, CD81, and TSG101 were positively expressed, while APOB and GRP94 were not expressed in the EV suspension (**Supplementary Figure 1E**). Therefore, EVs were successfully isolated from the BMSCs.

**Figure 2:**
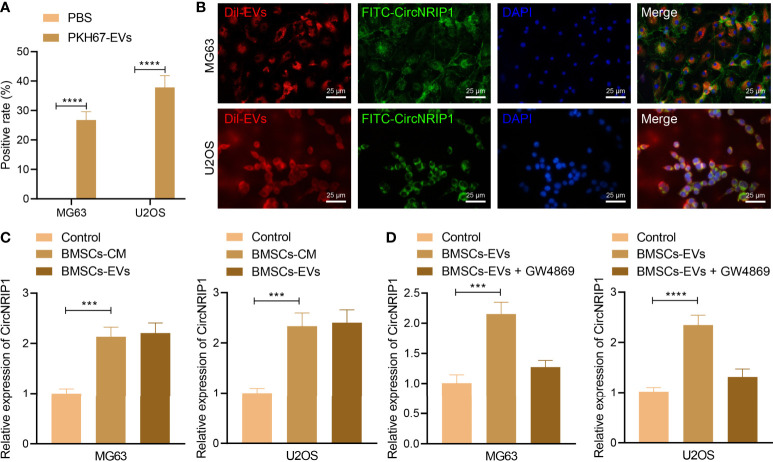
BMSC-EVs encapsulate circNRIP1 and deliver it to the recipient osteosarcoma cells. **(A)** Uptake of BMSC-derived EVs by osteosarcoma cells (MG63 and U2OS) observed under a confocal microscope, with PBS-treated cells serving as the negative control. **(B)** Co-localization of FITC-labeled circNRIP1 probe (green) and Dil-labeled EVs (red) in osteosarcoma cells. **(C)** Expression of circNRIP1 determined by RT-qPCR in the osteosarcoma cells co-cultured with BMSC-CM or BMSC-EVs (the volume of the BMSC-CM co-cultured with the cells was the same with the volume of the CM where BMSC-EVs had been isolated, and the CM used for BMSCs was DMEM). **(D)** The expression of circNRIP1 in osteosarcoma cells (MG63 and U2OS) measured by RT-qPCR after treatment with BMSC-EVs alone or in combination with EV secretion inhibitor GW4869 (the co-culture was performed in DMEM). ***p < 0.001. ****p < 0.0001. Data are shown as mean ± standard deviation of three technical replicates. Data between two groups were analyzed by unpaired *t*-test, while those among multiple groups were compared with one-way ANOVA, followed by Tukey’s *post hoc* tests.

Under a confocal microscope, we observed that a large number of BMSC-EVs entered into osteosarcoma cells and distributed around the nucleus, while no green fluorescence was found in osteosarcoma cells only treated with PBS (**Figure 2A**). The data following analysis utilizing the EVsrbase database indicated the presence of circNRIP1 in circulating EVs and in a variety of cells. Furthermore, we used FITC-circNRIP1 (green) probe to label the circNRIP1 in osteosarcoma cells that had been co-cultured with Dil-labeled EVs (red). As a result, there was co-localization of circNRIP1 and EVs in osteosarcoma cells, indicating that osteosarcoma cells had internalized the circNRIP1-loaded BMSC-EVs (**Figure 2B**). Additionally, the results of RT-qPCR showed higher expression of circNRIP1 in the osteosarcoma cells cultured in BMSC-CM than that in the control cells, and the circNRIP1 expression exhibited no evident difference in the osteosarcoma cells co-cultured with BMSC-EVs and with BMSC-CM (**Figure 2C**). Moreover, GW4869 (an inhibitor of EV release) treatment was observed to abrogate the increase in circNRIP1 expression in BMSCs co-cultured with EVs (**Figure 2D**). The above data indicated that circNRIP1 in BMSCs could be encapsulated and transferred into osteosarcoma cells by EVs.

### circNRIP1 Loaded by BMSC-EVs Promotes the Malignant Phenotypes of Osteosarcoma Cells

**Figure 3:**
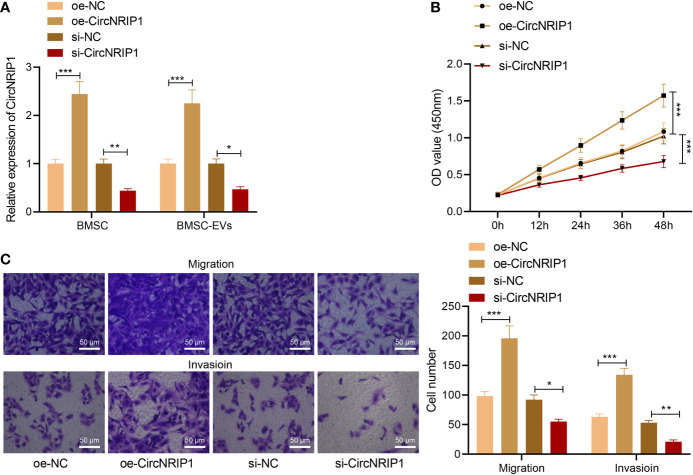
circNRIP1 loaded by BMSC-EVs promotes the malignant phenotypes of osteosarcoma cells. **(A)** Expression of circNRIP1 determined by RT-qPCR in EVs derived from BMSCs treated with oe-circNRIP1 or si-circNRIP1. **(B)** CCK-8 assay to detect the viability of MG63 cells co-cultured with EVs from BMSCs treated with oe-circNRIP1 or si-circNRIP1. **(C)** Transwell assay to detect the migration and invasion of MG63 cells co-cultured with EVs from BMSCs treated with oe-circNRIP1 or si-circNRIP1. *p < 0.05. **p < 0.01. ***p < 0.001. Data are shown as mean ± standard deviation of three technical replicates. Data among multiple groups were compared with one-way ANOVA, followed by Tukey’s *post hoc* tests. The repeated measures ANOVA with Bonferroni *post hoc* test was applied for the comparison of data at different time points.

We then moved to the investigation of the effects of BMSC-EV-loaded circNRIP1 in osteosarcoma. After co-culturing BMSC-EVs with MG63 osteosarcoma cells, the level of CircNRIP1 was upregulated in the cells, as revealed by RT-qPCR measurement (**Supplementary Figure 2**). Following that, we knocked down and overexpressed CircNRIP1 in BMSCs, respectively, and isolated corresponding BMSC-EVs. RT-qPCR results then indicated that EVs derived from BMSCs treated with oe-CircNRIP1 presented with elevated CircNRIP1 expression and that EVs from si-CircNRIP1-treated BMSCs presented with reduced CircNRIP1 expression (**Figure 3A**).

We then detected the malignant phenotypes of osteosarcoma cells in response to co-culture with BMSC-EVs with manipulated CircNRIP1. According to results from CCK-8 and Transwell assays, EVs from si-CircNRIP1-treated BMSCs led to repressed proliferative, migratory, and invasive potential of MG63 cells relative to the cells co-cultured with NC BMSC-EVs, while EVs from CircNRIP1 overexpression BMSCs, as compared with NC BMSC-EVs, exhibited promoting effects on the cell malignant behaviors (**Figures 3B, C**). Taken together, these results indicated that circNRIP1 loaded by BMSC-EVs promoted the malignant phenotypes of osteosarcoma cells.

### circNRIP1 Competitively Binds to 7miR-532-3p to Enhance the Malignant Phenotypes of Osteosarcoma Cells

**Figure 4:**
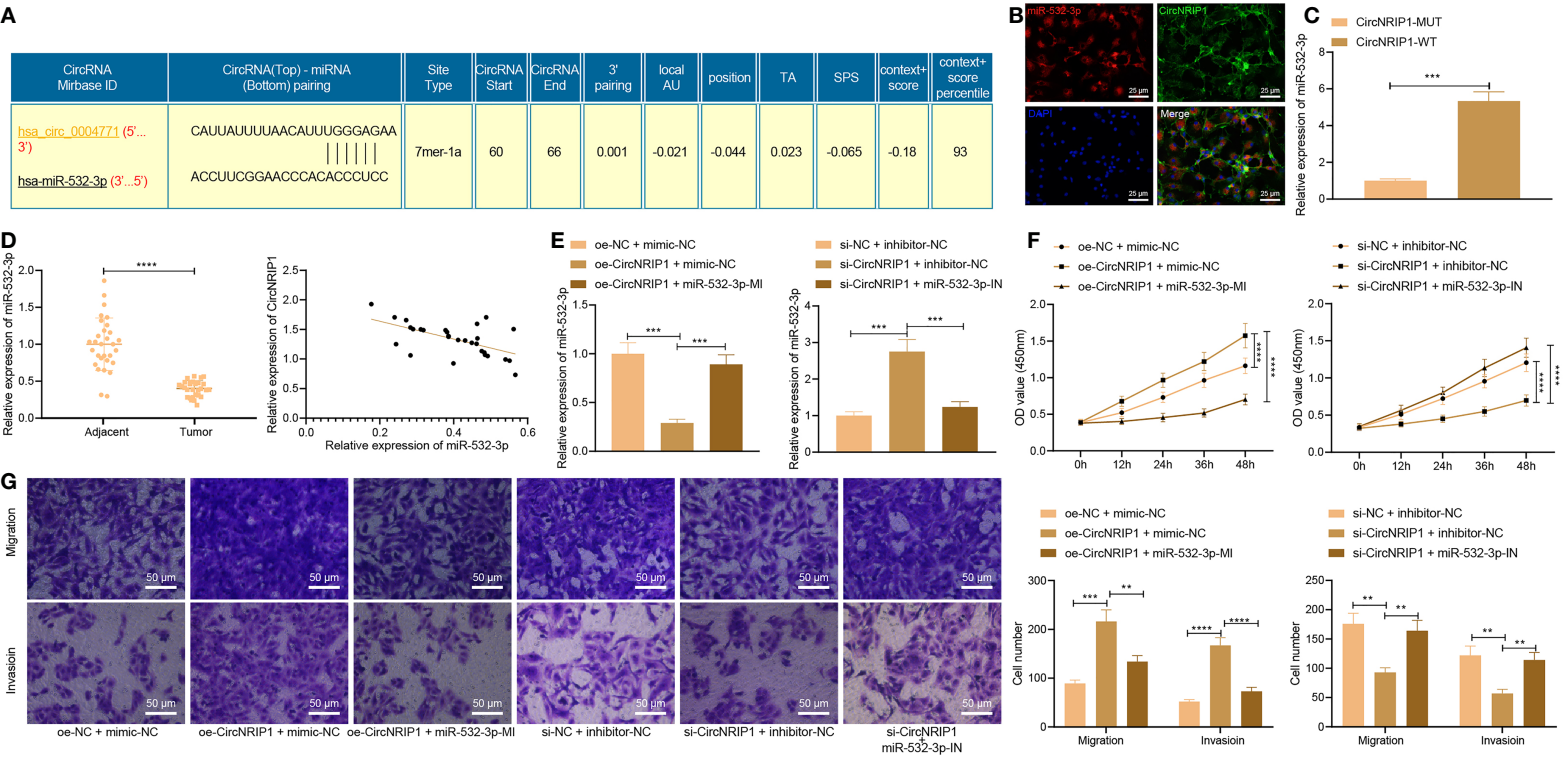
circNRIP1 competitively binding to miR-532-3p, accelerating the malignant phenotype of osteosarcoma cells. **(A)** The predicted complementary binding sites between circNRIP1 (also known as hsa_circ_0004771) and miR-532-3p by the StarBase database. **(B)** Co-localization of circNRIP1 (labeled by FITC, green) and miR-532-3p (labeled by Cy3, red) in MG63 cells measured by FISH. The nucleus was stained blue by DAPI. **(C)** miR-532-3p pulled down by circNRIP1-WT or circNRIP1-MUT assessed by RNA pull-down assay. **(D)** Expression of miR-532-3p determined by RT-qPCR in adjacent normal and osteosarcoma tissues (the left), and the correlation of miR-532-3p with circNRIP1 analyzed by Pearson’s correlation coefficient in osteosarcoma tissues (the right). **(E)** Expression of miR-532-3p determined by RT-qPCR in MG63 cells treated with oe-circNRIP1, si-circNRIP1, oe-circNRIP1 + miR-532-3p mimic, or si-circNRIP1 + miR-532-3p inhibitor. **(F)** Proliferation of MG63 cells treated with oe-circNRIP1, si-circNRIP1, oe-circNRIP1 + miR-532-3p mimic, or si-circNRIP1 + miR-532-3p inhibitor measured by CCK-8. **(G)** Migration and invasion of MG63 cells treated with oe-circNRIP1, si-circNRIP1, oe-circNRIP1 + miR-532-3p mimic, or si-circNRIP1 + miR-532-3p inhibitor measured by Transwell assay. **p < 0.01. ***p < 0.001. ****p < 0.0001. Data are shown as mean ± standard deviation of three technical replicates. Data between osteosarcoma tissues and adjacent normal tissues were compared using paired *t*-test and those between the other two groups by unpaired *t*-test. Data among multiple groups were compared with one-way ANOVA, followed by Tukey’s *post hoc* tests. The repeated measures ANOVA with Bonferroni *post hoc* test was applied for the comparison of data at different time points.

The downstream mechanism of circNRIP1 in osteosarcoma was our next focus. Complementary binding sites were predicted between circNRIP1 (also known as hsa_circ_0004771) and miR-532-3p by the StarBase database (**Figure 4A**). FISH results demonstrated that circNRIP1 and miR-532-3p were co-localized in MG63 cells (**Figure 4B**). The results of RNA pull-down assay showed more miR-532-3p pulled down by circNRIP1-WT (**Figure 4C**), which indicated that miR-532-3p had sequence specificity for the recognition of circNRIP1. Compared with the adjacent normal tissues, the expression of miR-532-3p was lower in osteosarcoma tissues, and an adverse correlation was evident in the circNRIP1 expression and miR-532-3p expression (**Figure 4D**). These results showed that miR-532-3p can be competitively bound by circNRIP1.

Furthermore, RT-qPCR results clarified a reduction of the miR-532-3p expression in MG63 cells overexpressing circNRIP1, while a contrary trend was noted upon simultaneous overexpression of circNRIP1 and miR-532-3p. circNRIP1 silencing resulted in upregulation of the miR-532-3p expression, whereas simultaneous silencing of circNRIP1 and miR-532-3p led to an opposite result (**Figure 4E**). The results of CCK-8 and Transwell assays unraveled an increase of proliferative, migratory, and invasive potential following circNRIP1 overexpression, while opposite trends were noted following additional miR-532-3p overexpression. In addition, circNRIP1 silencing caused impaired proliferative, migratory, and invasive potential which were negated following concomitant silencing of circNRIP1 and miR-532-3p (**Figures 4F, G**). These findings demonstrated that circNRIP1 could enhance the malignant phenotypes of osteosarcoma cells through competitive binding with miR-532-3p.

### circNRIP1 Upregulates the Expression of AKT3 by Competitively Binding to miR-532-3p

**Figure 5:**
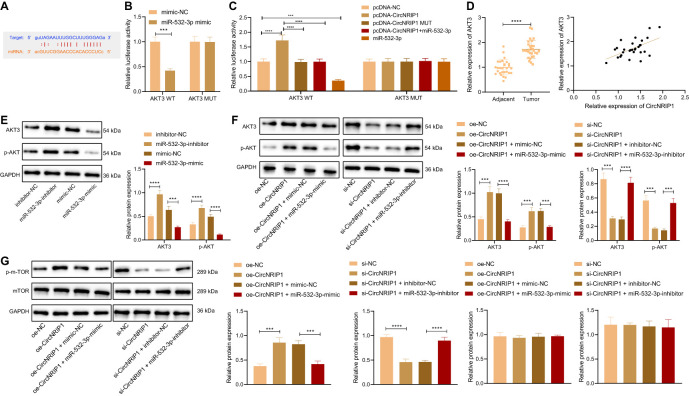
circNRIP1 increases the expression of AKT3 by competitively binding to miR-532-3p. **(A)** Predicted binding sites between miR-532-3p and AKT3 by the StarBase database. **(B)** Binding of miR-532-3p to AKT3 confirmed by dual-luciferase reporter assay in HEK-293T cells. **(C)** Binding between circNRIP1, miR-532-3p, and AKT3 confirmed by dual-luciferase reporter assay in HEK-293T cells. **(D)** Expression of AKT3 determined by RT-qPCR in adjacent normal and osteosarcoma tissues (the left), and the correlation of AKT3 with circNRIP1 analyzed by Pearson’s correlation coefficient in osteosarcoma tissues (the right). **(E)** Western blot analysis of AKT3 and p-AKT3 protein levels in MG63 cells transfected with miR-532-3p mimic or inhibitor. **(F)** Western blot analysis of AKT3 and p-AKT3 protein levels in MG63 cells treated with oe-circNRIP1, si-circNRIP1, oe-circNRIP1 + miR-532-3p mimic, or si-circNRIP1 + miR-532-3p inhibitor measured by Transwell assay. **(G)** Western blot measurement of p-mTOR and mTOR protein levels in MG63 cells in response to circNRIP1 overexpression/knockdown. ***p < 0.001. ****p < 0.0001. Data are shown as mean ± standard deviation of three technical replicates. Data between osteosarcoma tissues and adjacent normal tissues were compared using paired *t*-test and those between the other two groups by unpaired *t*-test. Data among multiple groups were compared with one-way ANOVA, followed by Tukey’s *post hoc* tests.

Then we studied the downstream regulatory mechanism of miR-532-3p. Prediction results of the StarBase database revealed the presence of binding sites between miR-532-3p and AKT3 (**Figure 5A**). Results of dual-luciferase reporter assay further presented that miR-532-3p could directly target the 3’-UTR of AKT3 (**Figure 5B**). In addition, overexpression of circNRIP1-WT increased the luciferase activity of pmirGLO-AKT3 vector, while ectopic expression of miR-532-3p reversed this increase (**Figure 5C**).

RT-qPCR data presented an enhancement of AKT3 mRNA expression in osteosarcoma tissues. Meanwhile, Pearson’s correlation coefficient revealed a positive correlation between circNRIP1 and AKT3 in osteosarcoma tissues (**Figure 5D**). Western blot analysis suggested that miR-532-3p overexpression downregulated the protein expression of AKT3 and p-AKT in MG63 cells, while miR-532-3p inhibition abolished the downregulation (**Figure 5E**). Furthermore, as shown in **Figure 5F**, the elevated AKT3 and p-AKT protein expression induced by circNRIP1 overexpression alone could be reversed by simultaneous overexpression of circNRIP1 and miR-532-3p, and the reduced AKT3 and p-AKT protein expression triggered by circNRIP1 silencing was negated by simultaneous silencing of circNRIP1 and miR-532-3p. Moreover, Western blot measurement of p-mTOR, a downstream effector of AKT, showed that circNRIP1 overexpression led to the upregulated protein level of p-mTOR, and circNRIP1 knockdown led to the opposite; also, additional restoration of miR-532-3p reversed the upregulation of p-mTOR induced by circNRIP1 overexpression alone, and the downregulation caused by circNRIP1 knockdown was reversed in response to miR-532-3p inhibition. The protein level of mTOR showed no obvious changes in response to the aforementioned treatments (**Figure 5G**). In summary, circNRIP1 had the potential to elevate the expression of AKT3 by competitively binding to miR-532-3p and further to modulate downstream molecules of AKT3.

### circNRIP1 Promotes Activation of the PI3K/AKT Signaling Pathway by Disrupting miR-532-3p-Mediated AKT3 Inhibition

**Figure 6:**
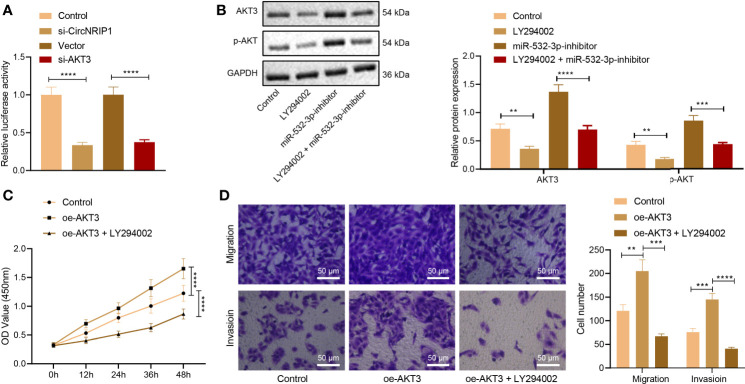
circNRIP1 induces activation of the PI3K/AKT signaling pathway *via* blockade of miR-532-3p-mediated AKT3 inhibition, thus facilitating the malignant phenotype of osteosarcoma cells. **(A)** The luciferase activity of the PI3K/AKT signaling pathway-related genes determined by dual-luciferase reporter assay upon knockdown of circNRIP1 or AKT3. **(B)** Western blot analysis of AKT3 and p-AKT3 protein levels in MG63 cells treated with oe-AKT3 or combined with LY294002. **(C)** Proliferation of MG63 cells treated with oe-AKT3 or combined with LY294002 measured by CCK-8. **(D)** Migration and invasion of MG63 cells treated with oe-AKT3 or combined with LY294002 measured by Transwell assay. **p < 0.01. ***p < 0.001. ****p < 0.0001. Data are shown as mean ± standard deviation of three technical replicates. Data among multiple groups were compared with one-way ANOVA, followed by Tukey’s *post hoc* tests. The repeated measures ANOVA with Bonferroni *post hoc* test was applied for the comparison of data at different time points.

Next, we focused on the downstream factors of AKT3. Analysis of the GSE41445 dataset revealed that differentially expressed genes were predominantly enriched in the PI3K/AKT signaling pathway, and AKT3 was at the core (**Figure 6A**). Additionally, the results of dual-luciferase reporter assay illustrated that the activity of PI3K/AKT signaling pathway was decreased upon knockdown of circNRIP1 or AKT3 (**Figure 6A**).

Western blot analysis indicated that treatment with LY294002 (the PI3K/AKT inhibitor) downregulated AKT3 protein expression in MG63 cells, while miR-532-3p inhibition brought about an opposite result. Combined treatment with LY294002 and miR-532-3p-inhibitor led to a decline in AKT3 and p-AKT protein expression (**Figure 6B**). The aforementioned results suggested that circNRIP1 activated the PI3K/AKT signaling pathway by elevating AKT3.

CCK-8 and Transwell assay data displayed enhanced proliferative, migratory, and invasive potential of MG63 cells in the presence of AKT3 overexpression, whereas a decline was witnessed following treatment with both oe-AKT3 and LY294002 (**Figures 6C, D**). The above data demonstrated that circNRIP1 activated the PI3K/AKT signaling pathway by impairing miR-532-3p-mediated AKT3 inhibition and aggravated osteosarcoma.

### circNRIP1-Containing BMSC-EVs Facilitate Tumor Growth *In Vivo*

**Figure 7:**
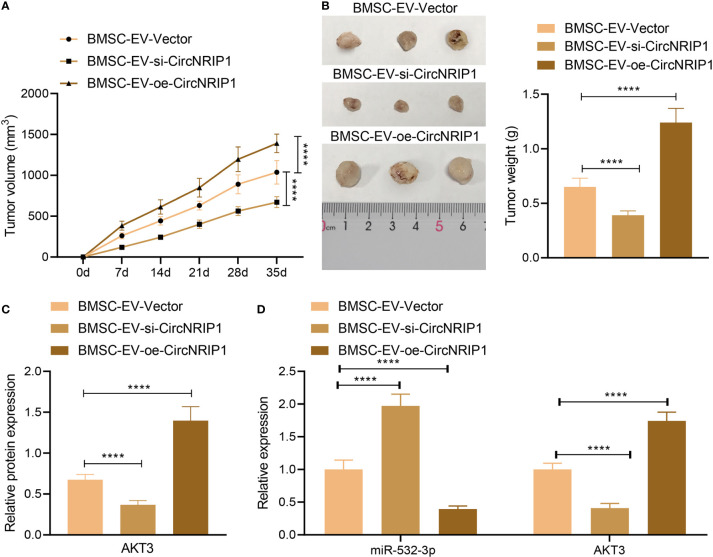
circNRIP1 boosts the growth of tumors *in vivo.* **(A)** Quantification of tumor volume of mice treated with si-circNRIP1 or oe-circNRIP1 for 5 weeks. **(B)** Tumor weight of mice treated with si-circNRIP1 or oe-circNRIP1 measured after euthanasia. **(C)** Western blot analysis of AKT3 protein in tumor tissues of mice treated with si-circNRIP1 or oe-circNRIP1. **(D)** Expression of AKT3 and miR-532-3p determined by RT-qPCR in tumor tissues of mice treated with si-circNRIP1 or oe-circNRIP1. N = 8 for mice following each treatment. ****p < 0.0001. Data are shown as mean ± standard deviation of three technical replicates. Data among multiple groups were compared with one-way ANOVA, followed by Tukey’s *post hoc* tests. The repeated measures ANOVA with Bonferroni *post hoc* test was applied for the comparison of data at different time points.

Finally, to characterize the effect of circNRIP1 on the osteosarcoma *in vivo*, we established a xenograft tumor model in nude mice and performed *in vivo* gain- and loss-of-function assays. According to the results, tumor growth was slowed down in response to si-circNRIP1-containing BMSC-EVs, as reflected by decreased tumor volume and weight, and accelerated in response to oe-circNRIP1-containing BMSC-EVs (**Figures 7A, B**). In addition, the mRNA and protein expression of AKT3 was reduced, and the miR-532-3p expression was elevated in tumor tissues of mice treated with si-circNRIP1, and oe-circNRIP1 treatment led to opposite results (**Figures 7C, D**). Taken together, these lines of evidence indicated that BMSC-EV-enclosed circNRIP1 could augment tumor growth *in vivo*.

## Discussion

Many studies have reported the potential application of EV-encapsulated circRNAs in the clinic as new biomarkers and therapeutic targets in different cancers (28, 29). The findings collected from this study supported the promoting effect of circNRIP1 encapsulated by BMSC-EVs on the progression of osteosarcoma *via* regulating the miR-532-3p/AKT3/PI3K/AKT pathway.

EVs derived from various types of cells exhibit distinct RNA profiles, which are essential cargoes in regulation of the hallmark of cancer and in reciprocal crosstalk within tumor cells, thus modulating the development and progression of cancer (30). circNRIP1 can be transmitted by EVs to gastric cancer cells where it promotes the malignant phenotypes of gastric cancer cells *in vitro* as well as stimulating tumor metastasis *in vivo* (31). Our initial results provided evidence suggesting that BMSC-EVs could encapsulate circNRIP1 and deliver it to the recipient osteosarcoma cells, where circNRIP1 exerted tumor-promoting effects. Emerging evidence indicates that circNRIP1 is upregulated in cancer tissues and cell lines and that they can augment cancer cell proliferation, migration, and invasion, such as cervical cancer and gastric cancer (26, 32). The present study revealed that circNRIP1 was highly expressed in osteosarcoma tissues and cells and was capable of stimulating the malignant phenotypes of osteosarcoma cells. Therefore, circNRIP1 delivered by BMSC-EVs may be useful as a potential prognostic biomarker and therapeutic target in osteosarcoma patients. However, due to the available literature, further investigation is a prerequisite.

The subsequent finding in the current study demonstrated that the promoting effect of circNRIP1 on the malignant phenotype of osteosarcoma cells was related to the upregulation of the miR-532-3p target AKT3 gene by competitively binding to miR-532-3p. Studies have confirmed that circNRIP1 can act as miRNA sponges and thus reduce their regulatory effects on the target mRNAs; for instance, circNRIP1 acts as a molecular sponge of miR-339-5p and then positively regulates the expression of the miR-339-5p target CDC25A (33). Additionally, circNRIP1 inhibits the expression of miR-653 by functioning as the competing endogenous RNA to sponge miR-653, thus weakening the inhibiting effect of miR-653 on the expression of its target ZEB2 (34). Moreover, a previous study found downregulated miR-532-5p in osteosarcoma tissues and cell lines and that miR-532-5p upregulation could suppress osteosarcoma cell growth and metastatic ability *in vitro*, as well as retarding osteosarcoma cell growth *in vivo* (20). Meanwhile, AKT3 has been detected to be increased in human osteosarcoma tissues and cells, and conversely, its reduction by miR-485-3p results in repressed cancer cell proliferation, migration, and invasion (35), which is very much in accordance to our results. These results suggested that the circNRIP1/miR-532-3p/AKT3 axis may be a promising prognostic marker and therapeutic target for osteosarcoma.

Further analysis exhibited that circNRIP1 could augment activation of the PI3K/AKT signaling pathway by disrupting miR-532-3p-mediated AKT3 inhibition. Studies have shown the promoting role of circRNAs in the activation of PI3K/AKT signaling pathway (36, 37), but the potential role of circNRIP1 on this pathway remains elucidated. Consistently, miR-532 overexpression has been reported to inhibit activation of the PI3K/AKT signaling pathway in colorectal cancer *in vitro* and *in vivo* (38). The positive correlation between AKT3 and the PI3K/AKT signaling pathway has been well-established (39, 40). Blockade of the PI3K/AKT signaling pathway by dammarenediol contributes to a decline in the viability of osteosarcoma cells and their metastatic potential (41). In addition, circ_0001785 has been reported to decrease the apoptosis of osteosarcoma cells through activation of the PI3K/Akt/mTOR pathway (42).

**Figure 8:**
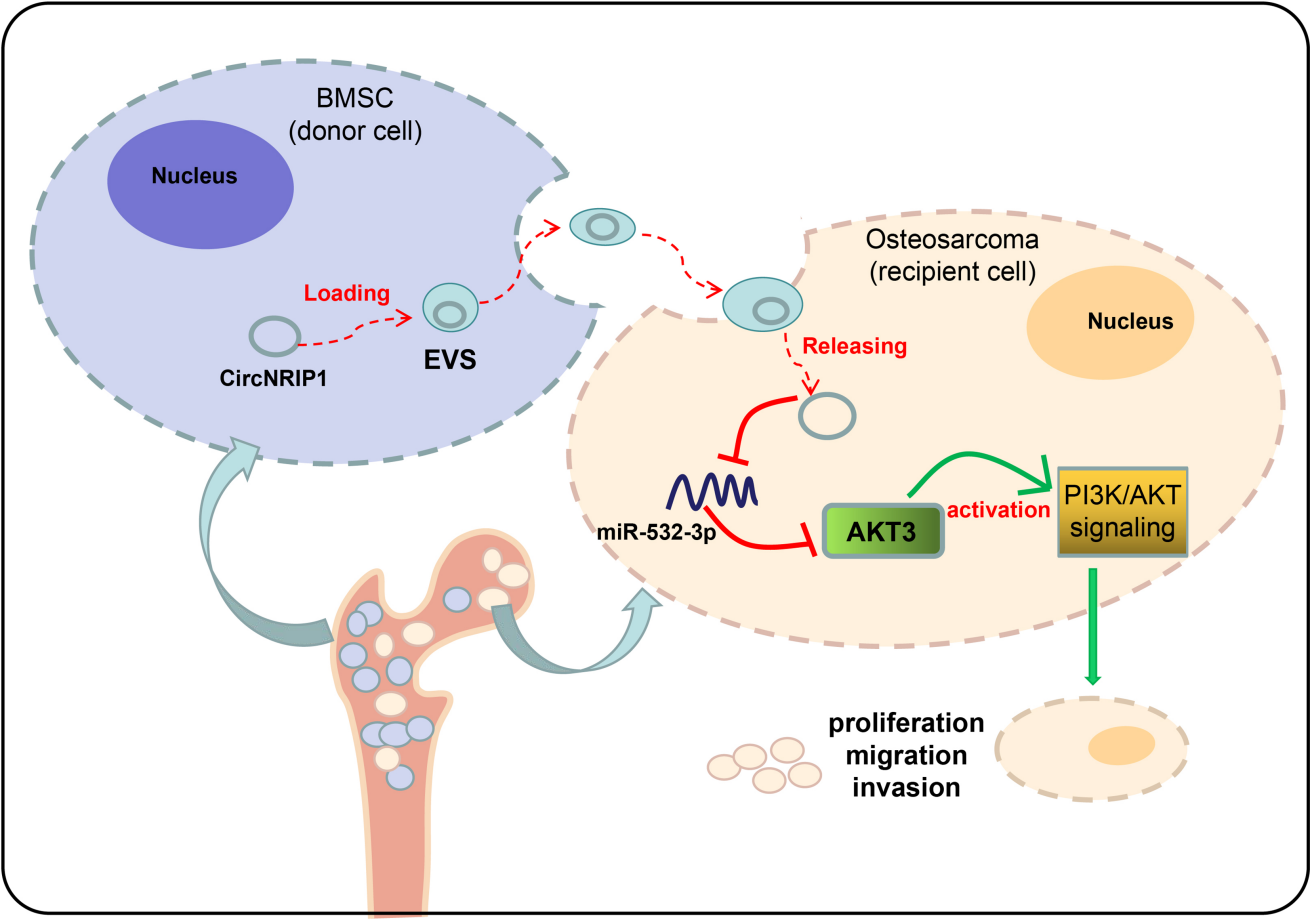
Underlying mechanism of circNRIP1 encapsulated by BMSC-EVs in osteosarcoma. BMSC-EVs can transfer circNRIP1 to osteosarcoma cells and promote the progression of osteosarcoma by regulating the miR-532-3p/AKT3/PI3K/AKT axis.

Overall, our study indicates that BMSC-EVs can transfer circNRIP1 to osteosarcoma cells and promote the progression of osteosarcoma by regulating the miR-532-3p/AKT3/PI3K/AKT axis (**Figure 8**). This novel axis can provide better understanding of the malignancy of osteosarcoma, which may further aid in the development of early detection molecular markers and risk stratification methods to improve the clinical care of osteosarcoma patients.

## Data Availability Statement

The original contributions presented in the study are included in the article/**Supplementary Material**. Further inquiries can be directed to the corresponding author.

## Ethics Statement

The studies involving human participants were reviewed and approved by the Ethics Committee of First Hospital of Harbin Medical University. The patients/participants provided their written informed consent to participate in this study. The animal study was reviewed and approved by the Animal Ethics Committee of First Hospital of Harbin Medical University.

## Author Contributions

ZS and KW designed the study. ZS, KW, YX, and XY collated the data, carried out data analyses and produced the initial draft of the manuscript. YX and XY contributed to drafting the manuscript. All authors contributed to the article and approved the submitted version.

## Conflict of Interest

The authors declare that the research was conducted in the absence of any commercial or financial relationships that could be construed as a potential conflict of interest.

## Publisher’s Note

All claims expressed in this article are solely those of the authors and do not necessarily represent those of their affiliated organizations, or those of the publisher, the editors and the reviewers. Any product that may be evaluated in this article, or claim that may be made by its manufacturer, is not guaranteed or endorsed by the publisher.

## Supplementary Material

Supplementary Figure 1 Isolation and characterization of EVs from BMSCs. **(A)** Identification of the phenotype of BMSCs by flow cytometry. **(B)** Morphological characterization of BMSCs observed using a light microscope. **(C)** Morphological characterization of the EVs isolated from the BMSCs observed using a TEM. **(D)** The size distribution of EVs analyzed by NTA. **(E)** Western blot analysis of EV surface maker proteins ALIX, CD81, TSG101, APOB, and GRP94. ****p* < 0.001. *****p* < 0.0001. Data are shown as mean ± standard deviation of three technical replicates. Data between two groups were analyzed by unpaired *t*-test.

Supplementary Figure 2 Expression of circNRIP1 determined by RT-qPCR in MG63 cells co-cultured with BMSC-EVs. After co-culturing BMSC-EVs with MG63 osteosarcoma cells, the level of CircNRIP1 was upregulated in the cells. ***p* < 0.01. Data are shown as mean ± standard deviation of three technical replicates. Data between two groups were analyzed by unpaired *t*-test.
