# Supplementary figures and images for: CircNRIP1 Encapsulated by Bone Marrow Mesenchymal Stem Cell–Derived Extracellular Vesicles Aggravates Osteosarcoma by Modulating the miR-532-3p/AKT3/PI3K/AKT Axis
Source: Front Oncol. 2021 Sep 29;11:658139. doi: 10.3389/fonc.2021.658139 (PMC8511523; doi:10.3389/fonc.2021.658139)

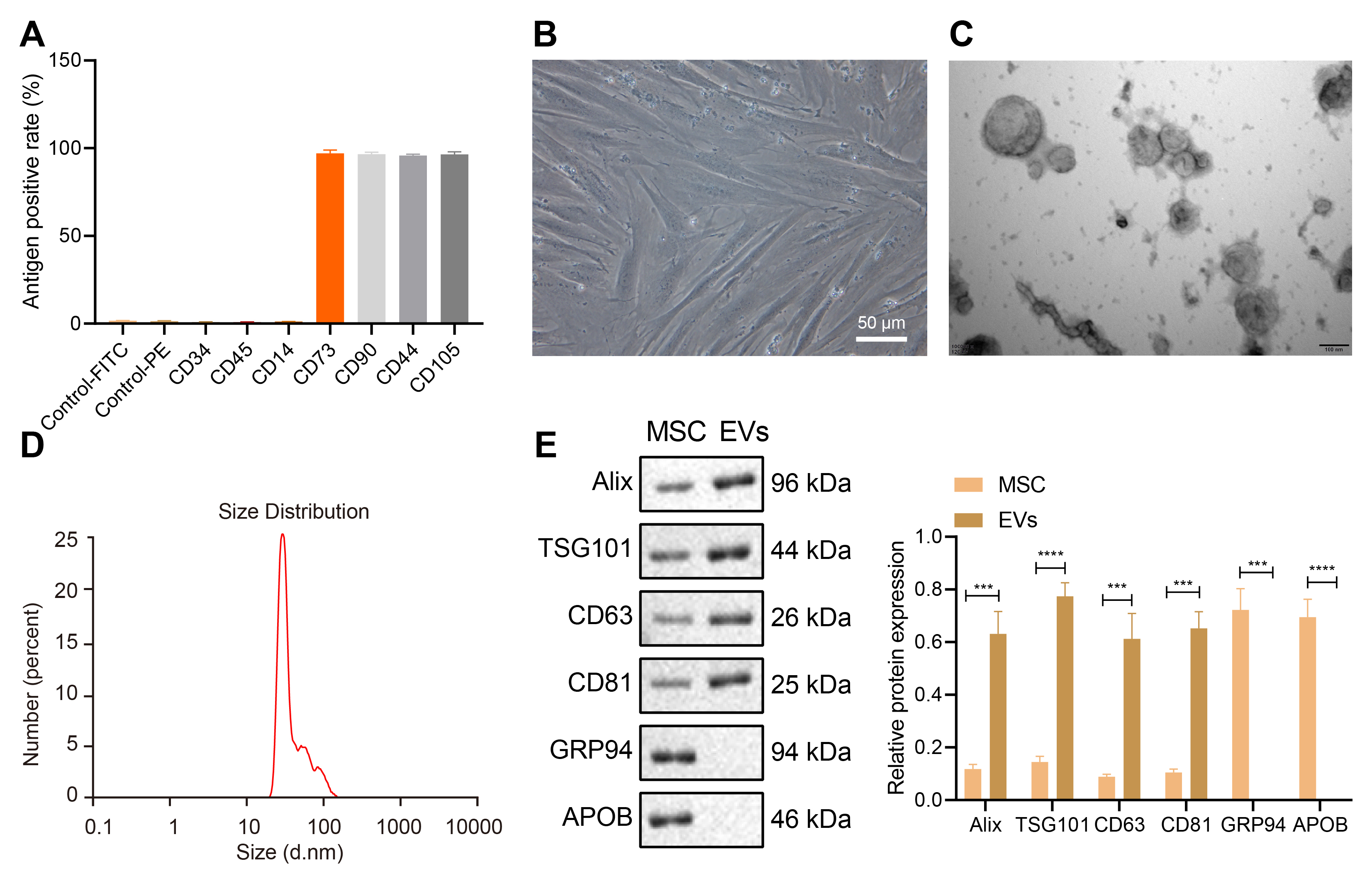

Supplement: Supplementary Figure 1 — Isolation and characterization of EVs from BMSCs. (A) Identification of the phenotype of BMSCs by flow cytometry. (B) Morphological characterization of BMSCs observed using a light microscope. (C) Morphological characterization of the EVs isolated from the BMSCs observed using a TEM. (D) The size distribution of EVs analyzed by NTA. (E) Western blot analysis of EV surface maker proteins ALIX, CD81, TSG101, APOB, and GRP94. ***p < 0.001. ****p < 0.0001. Data are shown as mean ± standard deviation of three technical replicates. Data between two groups were analyzed by unpaired t-test. [file Image_1.jpeg]

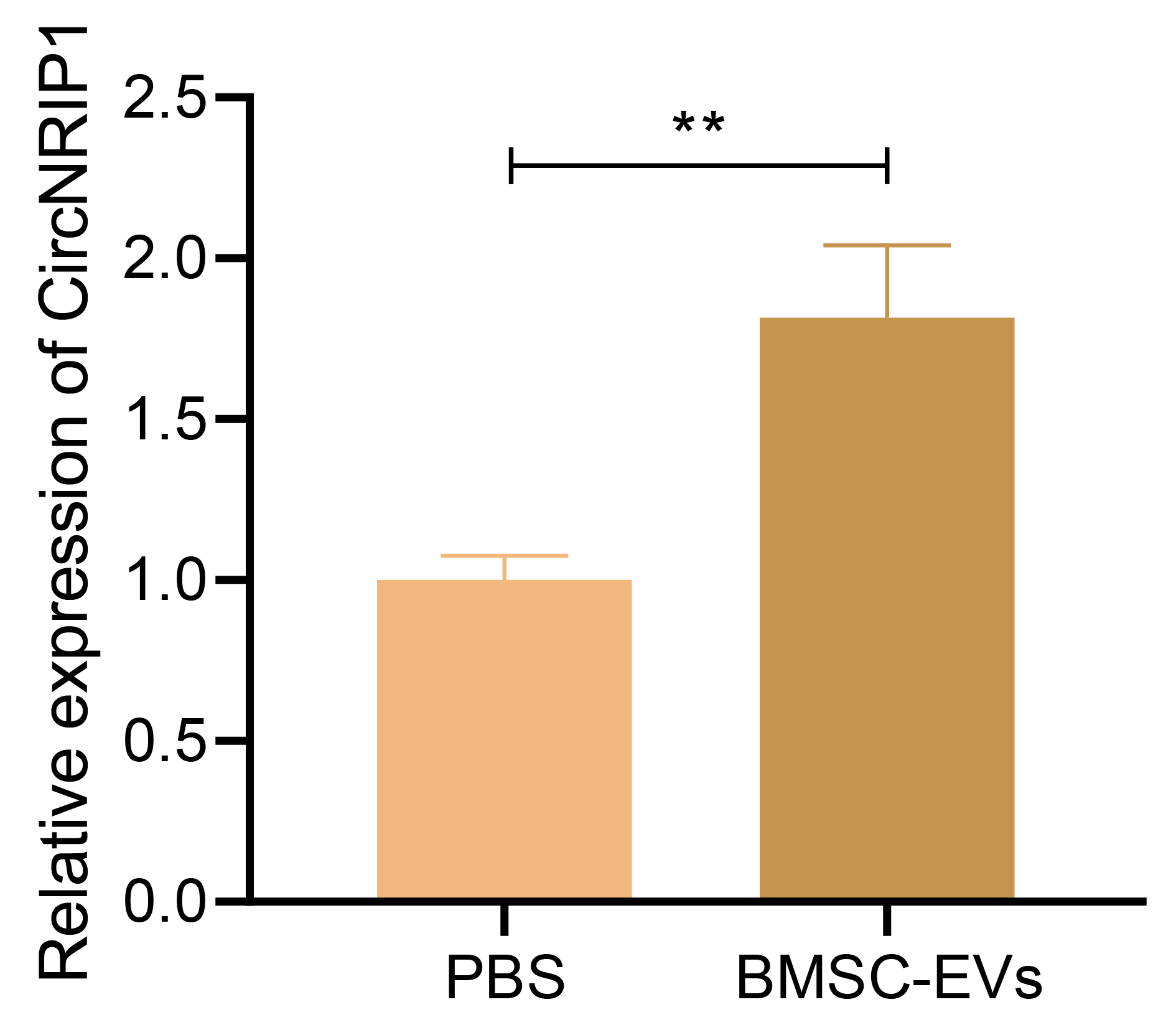

Supplement: Supplementary Figure 2 — Expression of circNRIP1 determined by RT-qPCR in MG63 cells co-cultured with BMSC-EVs. After co-culturing BMSC-EVs with MG63 osteosarcoma cells, the level of CircNRIP1 was upregulated in the cells. **p < 0.01. Data are shown as mean ± standard deviation of three technical replicates. Data between two groups were analyzed by unpaired t-test. [file Image_2.jpeg]
