# Supplementary material for: CircNRIP1 Encapsulated by Bone Marrow Mesenchymal Stem Cell–Derived Extracellular Vesicles Aggravates Osteosarcoma by Modulating the miR-532-3p/AKT3/PI3K/AKT Axis
Source: Front Oncol. 2021 Sep 29;11:658139. doi: 10.3389/fonc.2021.658139 (PMC8511523; doi:10.3389/fonc.2021.658139)
Supplement: Supplementary file 3 [file Table_1.docx]

**Supplementary table 1** Primer sequences for reverse transcription quantitative polymerase chain reaction

| Gene | Primer sequence |
| --- | --- |
| circNRIP1 | Forward 5’-ACAGCCAGAAGATGCACACTTG-3’ |
|  | Reverse 5’-TGGGGCTACGAGATAAAGGAGA-3’ |
| AKT3 | Forward 5’-TCAACAACTTTTCAGTGGCAAAA-3’ |
|  | Reverse 5’-ATTCTTCCCTTTCCTCTGGAGTATC-3’ |
| β-actin | Forward 5’-CTCGCCTTTGCCGATCC-3’ |
|  | Reverse 5’-TCTCCATGTCGTCCCAGTTG-3’ |
| U6 | Forward 5’-GCTTCGGCAGCACATATACTAAAAT-3' |
|  | Reverse 5’-CGCTTCACGAATTTGCGTGTCAT-3’ |
| GAPDH | Forward 5’-AGGTGAAGGTCGGAGTCAAC-3' |
|  | Reverse 5’-GCTCCTGGAAGATGGTGATGG-3’ |

**Note:** GAPDH, glyceraldehyde-3-phosphate dehydrogenase

**Supplementary table 2** Sequences of AKT3-3'-UTR-WT and AKT3-3'-UTR MUT

| Gene | Sequence |
| --- | --- |
| AKT3-3'-UTR-WT | 5’-UAGAAUUUGGCUUUGGGAG-3’ |
| AKT3-3'-UTR-MUT | 5’-UCGAAGCCAACGUGCUUGA-3’ |

**Note:** WT, wild type; MUT, mutant

**Supplementary table 3** Sequences of oe/sh-circNRIP1 used in mice experiment

| Gene | Sequence |
| --- | --- |
| oe-circNRIP1 | ATGACTCATGGAGAAGAGCTTGGCTCTGATGTGCACCAGGATTCTATTGTTTTAACTTACCTAGAAGGATTACTAATGCATCAGGCAGCAGGGGGATCAGGTACTGCCGTTGACAAAAAGTCTGCTGGGCATAATGAAGAGGATCAGAACTTTAACATTTCTGGCAGTGCATTTCCCACCTGTCAAAGTAATGGTCCAGTTCTCAATACACATACATATCAGGGGTCTGGCATGCTGCACCTCAAAAAAGCCAGACTGTTGCAGTCTTCTGAGGACTGGAATGCAGCAAAGCGGAAGAGGCTGTCTGATTCTATCATGAATTTAAACGTAAAGAAGGAAGCTTTGCTAGCTGGCATGGTTGACAGTGTGCCTAAAGGCAAACAGGATAGCACATTACTGGCCTCTTTGCTTCAGTCATTCAGCTCTAGGCTGCAGACTGTTGCTCTGTCACAACAAATCAGGCAGAGCCTCAAGGAGCAAGGATATGCCCTCAGTCATGATTCTTTAAAAGTGGAGAAGGATTTAAGGTGCTATGGTGTTGCATCAAGTCACTTAAAAACTTTGTTGAAGAAAAGTAAAGTTAAAGATCAAAAGCCTGATACGAATCTTCCTGATGTGACTAAAAACCTCATCAGAGATAGGTTTGCAGAGTCTCCTCATCATGTTGGACAAAGTGGAACAAAGGTCATGAGTGAACCGTTGTCATGTGCTGCAAGATTACAGGCTGTTGCAAGCATGGTGGAAAAAAGGGCTAGTCCTGCCACCTCACCTAAACCTAGTGTTGCTTGTAGCCAGTTAGCATTACTTCTGTCAAGCGAAGCCCATTTGCAGCAGTATTCTCGAGAACACGCTTTAAAAACGCAAAATGCAAATCAAGCAGCAAGTGAAAGACTTGCTGCTATGGCCAGATTGCAAGAAAATGGCCAGAAGGATGTTGGCAGTTACCAGCTCCCAAAAGGAATGTCAAGCCATCTTAATGGTCAGGCAAGAACATCATCAAGCAAACTGATGGCTAGCAAAAGTAGTGCTACAGTGTTTCAAAATCCAATG  GGTATCATTCCTTCTTCCCCTAAAAATGCAGGTTATAAGAACTCACTGGAAAGAAACAATATAAAACAAGCTGCTAACAATAGTTTGCTTTTACATCTTCTTAAAAGCCAGACTATACCTAAGCCAATGAATGGACACAGTCACAGTGAGAGAGGAAGCATTTTTGAGGAAAGTAGTACACCTACAACTATTGATGAATATTCAGATAACAATCCTAGTTTTACAGATGACAGCAGTGGTGATGAAAGTTCTTATTCCAACTGTGTCCCATAGACTTGTCTTGCAAACACCGAACTGAAAAATCAGAATCTGACCAACCTGTTTCCCTGGATAACTTCACTCAATCCTTGCTAAACACTTGGGATCCAAAAGTCCCAGATGTAGATATCAAAGAAGATCAAGATACCTCAAAGAATTCTAAGCTAAACTCACACCAGAAAGTAACACTTCTTCAATTGCTACTTGGCCATAAGAATGAAGAAAATGTAGAAAAAAACACCAGCCCTCAGGGAGTACACAATGATGTGAGCAAGTTCAATACACAAAATTATGCAAGGACTTCTGTGATAGAAAGCCCCAGTACAAATCGGACTACTCCAGTGAGCACTCCACCTTTACTTACATCAAGCAAAGCAGGGTCTCCCATCAATCTCTCTCAACACTCTCTGGTCATCAAATGGAATTCCCACCATATGTCTGCAGTACTCAGTCTGAAAAGCTAACAAATACTGCATCTAACCACTCAATGGACCTTACAAAAAGCAAAGACCCACCAGGAGAGAAACCAGCCCAAAATGAAGGTGCACAGAACTCTGCAACGTTTAGTGCCAGTAAGCTGTTACAAAATTTAGCACAATGTGGAATGCAGTCATCCATGTCAGTGGAAGAGCAGAGACCCAGCAAACAGCTGTTAACTGGAAACACAGATAAACCGATAGGTATGATTGATAGATTAAATAGCCCTTTGCTCTCAAATAAAACAAATGCAGTTGAAGAAAATAAAGCATTTAGTAGTCAACCAACAGGTCCTGAACCAGGGCTTTCTGGTTCTGAAATAGAAAATCTGCTTGAAAGACGTACTGTCCTCCAGTTGCTCCTGGGGAACCCCAACAAAGGGAAGAGTGAAAAAAAAGAGAAAACTCCCTTAAGAGATGAAAGTACTCAGGAACACTCAGAGAGAGCTTTAAGTGAACAAATACTGATGGTGAAAATAAAATCTGAGCCTTGTGATGACTTACAAATTCCTAACACAAATGTGCACTTGAGCCATGATGCTAAGAGTGCCCCATTCTTGGGTATGGCTCCTGCTGTGCAGAGAAGCGCACCTGCCTTACCAGTGTCCGAAGACTTTAAATCGGAGCCTGTTTCACCTCAGGATTTTTCTTTCTCCAAGAATGGTCTGCTAAGTCGATTGCTAAGACAAAATCAAGATAGTTACCTGGCAGATGATTCAGACAGGAGTCACAGAAATAATGAAATGGCACTTCTAGAATCAAAGAATCTTTGCATGGTCCCTAAGAAAAGGAAGCTTTATACTGAGCCATTAGAAAATCCATTTAAAAAGATGAAAAACAACATTGTTGATGCTGCAAACAATCACAGTGCCCCAGAAGTACTGTATGGGTCCTTGCTTAACCAGGAAGAGCTGAAATTTAGCAGAAATGATCTTGAATTTAAATATCCTGCTGGTCATGGCTCAGCCAGCGAAAGTGAACACAGGAGTTGGGCCAGAGAGAGCAAAAGCTTTAATGTTCTGAAACAGCTGCTTCTCTCAGAAAACTGTGTGCGAGATTTGTCCCCGCACAGAAGTAACTCTGTGGCTGACAGTAAAAAGAAAGGACACAAAAATAATGTGACCAACAGCAAACCTGAATTTAGCATTTCTTCTTTAAATGGACTGATGTACAGTTCCACTCAGCCCAGCAGTTGCATGGATAACAGGACATTTTCATACCCAGGTGTAGTAAAAACTCCTGTGAGTCCTACTTTCCCTGAGCACTTGGGCTGTGCAGGGTCTAGACCAGAATCTGGGCTTTTGAATGGGTGTTCCATGCCCAGTGAGAAAGGACCCATTAAGTGGGTTATCACTGATGCGGAGAAGAATGAGTATGAAAAAGACTCTCCAAGATTGACCAAAACCAACCCAATACTATATTACATGCTTCAAAAAGGAGGCAATTCTGTTACCAGTCGAGAAACACAAGACAAGGACATTTGGAGGGAGGCTTCATCTGCTGAAAGTGTCTCACAGGTCACAGCCAAAGAAGAGTTACTTCCTACTGCAGAAACGAAAGCTTCTTTCTTTAATTTAAGAAGCCCTTACAATAGCCATATGGGAAATAATGCTTCTCGCCCACACAGCGCAAATGGAGAAGTTTATGGACTTCTGGGAAGCGTGCTAACGATAAAGAAAGAATCAGAATAA’ |
| si-circNRIP1 | 5’-GAAAGAATTTAACAGAGTATCA-3’ |
| Vector | 5’-CGCTCAAGCCCAGAGTCAGCAG-3’ |
